# Cognitive, behavioral, and psychological manifestations of COVID-19 in post-acute rehabilitation setting: preliminary data of an observational study

**DOI:** 10.1007/s10072-021-05653-w

**Published:** 2021-10-12

**Authors:** Silvia Bonizzato, Ada Ghiggia, Francesco Ferraro, Emanuela Galante

**Affiliations:** 1 Department of Psychology, University of Milan-Bicocca, Piazza dell’Ateneo Nuovo 1, Milan, Italy; 2 Department of Psychology, University of Turin, Via Verdi 10, 10124 Turin, Italy; 3 Neuro-Motor Rehabilitation Unit, Neuroscience Department, Azienda Socio Sanitaria Territoriale Di Mantova, via XXV Aprile 71, Bozzolo, Mantova, Italy

**Keywords:** COVID-19, Neuropsychological deficit, Psychological assessment, Behavioral alterations

## Abstract

Psychological, emotional, and behavioral domains could be altered in COVID-19 patients and measurement of variables within these domains seems to be mandatory. Neuropsychological assessment could detect possible cognitive impairment caused by COVID-19 and the choice of appropriate tools is an important question. Aim of this exploratory study was to verify the effectiveness of an assessment model for patients with COVID-19. Twelve patients were enrolled and tested with Mini-Mental State Examination (MMSE), Montreal Cognitive Assessment (MoCA), Anxiety and Depression Short Scale (AD-R), and the Neuropsychiatry Inventory (NPI), at the time of their entrance (T0) and discharge (T1) from a rehabilitative unit. Moreover, a follow-up evaluation after 3 months (T2) has been conducted on eight patients. Results showed that at baseline (T0), 58.3% of the patients reported a score below cut-off at MMSE and 50% at MoCA. Although a significant amelioration was found only in NPI scores, a qualitative improvement has been detected at all tests, except for MoCA scores, in the T0-T1 trend analysis. A one-way repeated measures analysis of variance showed a significant variation in AD-R depression score, considering the three-assessment time (T0, T1, and T2). The evaluation and tracking over time of the impact of COVID-19 on cognitive, psychological, and behavioral domains has relevant implications for rehabilitation and long-term assistance needs planning. The choice of assessment tools should consider patients vulnerability and match the best compromise among briefness, sensitivity, and specificity.

## Introduction

Severe acute respiratory syndrome coronavirus 2 (SARS-CoV-2) is the cause of the coronavirus disease 2019 (COVID-19), mainly characterized by respiratory illness. It emerged in Wuhan, China, in December 2019 and caused a pandemic outbreak, according to the World Health Organization (WHO).

In the past months, reports from different countries suggested that SARS-CoV-2 could directly and indirectly infect structures of the nervous system [1–4]. The central (CNS) and peripheral nervous system (PNS) involvement may be related to hypoxia and endothelial damage, uncontrollable immune reaction and inflammation, electrolyte imbalance, hypercoagulable state and disseminated intravascular coagulation, septic shock, and/or multiple organ failure [5, 6]. Moreover, the adhesion mechanism of SARS-CoV-2 to ACE2 receptors gains implications on blood pressure cerebral regulation and on the question about neurotropism of SARS-CoV-2 [7]. COVID-19 can complicate or co-exist with cerebrovascular disease [8] or other neurological disease like multiple sclerosis [9], Alzheimer’s disease [10], and Parkinson’s disease [7] suggesting CNS involvement and complex neurological clinical complications.

It has been suggested that, beside neurological symptoms, it is important monitoring potential late COVID-19 neurological sequelae like possible neuropsychological deficits [2, 11], as well as seen in other viral infection [11].

In recent studies [12–15], attention has been paid to low cognitive performance caused by COVID-19. Cognitive impairment has been put in relation with headache [11], inflammatory processes [13], clinical and metabolic alterations [14], and length of stay in intensive care unit [12]. To date, few studies evaluated over time neuropsychological deficits of COVID-19 patients. These studies showed a poor performance in attention and executive domain tests suggesting a dysexecutive syndrome related to COVID-19 [14, 15]. Only one study [16] proposed possible presence of focal neuropsychological disorders, such as agraphia and conduction aphasia, but the authors did not necessarily link them to the COVID-19. The importance of a specific neuropsychological rehabilitation has also been suggested [12, 15], highlighting the importance of investigating long-term cognitive functional trends in patients.

In addition, few authors emphasized the importance of monitoring and managing the possible behavioral and psychological consequences of neurobiological factors, traumatic experiences, and setting organization (i.e., social isolation, immobilization) [17, 18]. Psychological and behavioral manifestations should be considered in COVID-19 patients. Some studies showed that psychological symptoms are more prevalent than neurological ones [15, 19]. Moreover, previous studies of similar viral respiratory diseases, such as severe acute respiratory syndrome (SARS) during the 2002–2004 outbreak in China, indicated that behavioral alterations of infected individuals should not be ignored [20]. Studies on the mental status of COVID-19 patients show the presence of depression, anxiety, and possible post-traumatic stress disorders (PTSD) [21]. Correlation between physical condition and psychological morbidities is evident and has been highlighted in few studies [15, 22, 23].

The evaluation and tracking over time of the impact of COVID-19 on cognitive, psychological, and behavioral patient conditions have relevant implications for rehabilitation planning and long-term assistance needs. However, the choice of appropriate tools is still open to debate. In particular, concerning our experience, at the arrival in the post-acute rehabilitative setting, patients are physically and mentally vulnerable due to the relevant metabolic alterations, long immobilization, and social isolation to which they have been subjected. Therefore, instruments for a first evaluation should match the best compromise among briefness, sensitivity, and specificity.

Aim of this exploratory study was to verify the effectiveness of an assessment model for patients recovering from COVID-19, at the time of their entrance and discharge from the rehabilitative hospital (T0 and T1) and after 3 months (T2).

## Methods

### Participants

Twelve patients with COVID-19 were included in the study; they were transferred to the Physical Therapy Department of the Carlo Poma Hospital in Mantua (Italy) after hospitalization for COVID-19. The study was approved by the Institutional Ethic Committee and was conducted in accordance with the Declaration of Helsinki. All the participants gave their written informed consent to participate in the study.

All patients met COVID-19 diagnostic criteria and after the acute phase of the disease have been moved to the rehabilitative unit. No other inclusion criteria were requested. Exclusion criteria were disorders, which precluded answering to the tests, such as delirium, aphasia, or overt dementia. Six patients met severe pneumonia caused by COVID-19 infection, four were affected by stroke complicated by the infection, one met COVID-19 infection after an organ transplant, and one had some degree of memory impairment before contracting the virus. This last patient was already known to our Neuropsychology Unit for a previous evaluation, which has been considered as a baseline respect to the present assessment.

Patients were tested at the arrival (T0) and discharge from their rehabilitative hospitalization (T1, after about a month) with screening tools for global cognitive evaluation. Eight of them were also evaluated after 3 months (T2) to monitor the impact on cognitive-behavioral psychological profile over time. Four patients refuse to come back for follow up and were considered dropped out. Six of them were tested at T2 through an extended cognitive assessment, in addition to the screening tests in order to better detect the presence in the long term of possible mild impairment or deficit. The remaining two patients were too weak to complete the investigation with the extended cognitive profile.

During the rehabilitation, patients received an individually tailored multidisciplinary intervention (physical therapy, psychological support, cognitive stimulation, and occupational therapy). Due to infection limitations, such as movement restrictions, contact isolation, and oxygen dependence, cognitive stimulation interventions were limited in time (about 20 min per patients) and in content. General stimulation included temporal and spatial orientation and exercises of general attention and some executive functions (room and picture description, daily planning, verbal fluency).

### Assessment

MMSE (Mini-Mental State Examination) [24] and MoCA (Montreal Cognitive Assessment) [25] were selected as screening tests for global cognitive assessment. While the MMSE is universally used as a screening test for possible cortical dementia (this test covers the domains of temporal and spatial orientation, attention, memory, language, and visual-spatial abilities), the MoCA is more sensitive to detect impairment of executive functions, having subtests of attention shifting, concentration, abstraction, phonemic fluency, and subtests investigating the same cognitive domains as the MMSE.

An extended cognitive evaluation has been performed, in order to investigate memory, attention, executive functions, and verbal fluency domains, according to the neuropsychological clinical practice [26], which requires an extended assessment through the different domains. We selected the following tests: Digit and Corsi span forward and backward [27] for short-term and working verbal and visuo-spatial memory; Rey Auditory Verbal Learning (RAVL) [28]; Spatial Recall Test (SPART) [29] for respectively long-term verbal and visuo-spatial memory; Symbol Digit Modalities Test (SDMT) [30] for selective attention; Trail Making Test (TMT) [31] for attentional shift; Stroop Test [32]; and Frontal Assessment Battery (FAB) [33] for executive functions, such as processing speed, motor programming, inhibitory control and environmental autonomy, verbal abstraction, and phonemic fluency FAS [34] for language.

The possible presence of behavioral and psychological symptoms was investigated through a psychological interview and instruments selected to gather information on anxiety, depression (Anxiety and Depression Short Scale — AD-R) [35], and behavioral alterations (Neuropsychiatry Inventory — NPI) [36]. Score of the AD-R anxiety scale is considered critical when it is above the 80° percentile [35], while the score of AD-R depression scale is considered critical when is above the 90° percentile. Presence of any delusions or hallucinations, depression scores above six, disinhibition scores above four, and irritability score above two are to be taken into account at the NPI [36]. Trained psychologists administered tests and questionnaires.

### Statistical analysis

Statistical analyses were performed using the Statistical Package for Social Science — for Mac, Version 26.0 (SPSS Inc., Chicago, IL, USA).

Descriptive statistics were used to describe socio-demographic, cognitive, and psychological data; data that did not follow a normal distribution were described using medians and the lower and upper quartiles. Given the small sample size, Wilcoxon’s rank sum test was applied for comparison of the cognitive and psychological variables at baseline (T0) and at the time of hospital discharge (T1).

Different one-way repeated measures analysis of variance (ANOVA) were launched to determine whether there were any statistically significant differences between the score of the neuropsychological and behavioral variables at the three different times of evaluation. Where significant differences were observed, a Wilcoxon rank-test was applied to determine between what times these differences were observed.

For all analyses, *p* values < 0.05 were considered statistically significant.

## Results

### Demographic characteristics and testing results

Participants were 12 adults (mean age 71.33 ± 10.08 year; range 47–85), and 41.7% were females; education year was 7.25 ± 3.34 (range 4–13).

At T0, seven patients (58.3%) had a performance below cut-off at the MMSE and six (50%) at MoCA. Four patients (33.3%) obtained critical scores at the anxiety scale and two (16.6%) at the depression scale. Five subjects (41.66%) reported abnormal scores to NPI.

At discharge (T1), four patients (33.3%) obtained scores below cut-off at the MMSE, and six (50%) at the MoCA. Three patients (25%) had high anxiety level and only two (16.66%) reported behavioral symptoms by means of the NPI (see Table 1).

**Table 1:** Number and percentage of patients with test scores below the threshold values

Variables	T0 *N* = 12	T1 *N* = 12	T2 *N* = 8
MMSE	7 (58.3%)	4 (33.3%)	2 (25%)
MoCA	6 (50%)	6 (50%)	4 (50%)
AD-R, anxiety scale	4 (33.3%)	3 (25%)	0 (0%)
AD-R, depression scale	2 (16.6%)	0 (0%)	1 (8.3%)
NPI, total score	5 (41.66%)	2 (16.66%)	2 (25%)

Abbreviations: *MMSE*, Mini-Mental State Examination; *MoCA*, Montreal Cognitive Assessment; *AD-R*, Anxiety and Depression Short Scale; *NPI*, Neuropsychiatry Inventory Scale

Among the eight patients tested at T2, two (25%) had a poor performance at the MMSE, four (50%) at the MoCA, one patient (8.3%) presented depressive symptoms, and only two (25%) critical behavioral scores at the NPI. Two participants tested at T2 were too weak to approach the complete cognitive profile. Among remaining participants, five (62.5%) provided a performance below cut-off at the SDMT, and three (37.5%) to some memory test (RAVL, Corsi span backward, and SPART).

Data on neuropsychological assessment at T2 are reported in Table 2.

**Table 2:** Number and percentage of patients (*N* = 6) with neuropsychological test scores below the threshold values at the follow-up assessment (T2)

Memory	
Digit span forward	1 (12.5%)
Digit span backward	1 (12.5%)
RAVL-immediate recall	3 (37.5%)
RAVL-delayed recall	2 (25%)
Corsi span forward	1 (12.5%)
Corsi span backward	3 (37.5%)
SPART	3 (37.5%)
SPART-D	1 (12.5%)
Attention and executive functions	
SDMT	5 (62.5%)
TMT — A	2 (25%)
TMT — B	2 (25%)
TMT — B-A	2 (25%)
*Test di Stroop (Caffarra *et al*., 2002)*	
Stroop test — errors	2 (25%)
Stroop test — time	0 (0%)
FAB	2 (25%)
FAS	2 (25%)

Abbreviations: *RAVL-I*, Rey auditory verbal learning-immediate recall; *RAVL-D*, Rey auditory verbal learning-delayed recall; *SPART*, spatial recall test; *SPART-D*, spatial recall test-delayed; *SDMT*, symbol digit modalities test; *TMT*, trail making test; *FAB*, frontal assessment battery; *FAS*, phonemic fluency

### Trend of cognitive, psychological, and behavioral measures along time

Preliminary analysis was conducted to find possible differences in neuropsychological, psychological, and/or behavioral variables between T0 and T1 (Table 3). A significant decrease was found in NPI scores, *z* =  − 2.304, *p* < 0.05. No other significant median changes emerged at the Wilcoxon signed-rank test, neither in neuropsychological screening tests (MMSE: *z* =  − 0.366, *p* = 0.714; MoCA: *z* =  − 1.846, *p* = 0.065) nor in psychological measures (AD-R), for anxiety (*z* =  − 1.183, *p* = 0.237) or depression (*z* =  − 1.798, *p* = 0.072).

**Table 3:** Neuropsychological and behavioral tests, median (IQR), and Wilcoxon signed‐rank test between T0 and T1 (*N* = 12)

Variables	T0	T1	*p*-value*
MMSE, total score	22.5 (16.39-26.61)	25.5 (16.22–29.28)	0.714
MoCA, total score	14.5 (7.95–18.55)	13.0 (8.77–20.98)	0.065
AD-R, anxiety scale	21.5 (14.39–28.11)	17.5 (13.36–24.14)	0.237
AD-R, depression scale	4.0 (2.47–8.03)	1.5 (0.32–5.68)	0.072
NPI, total score	8.5 (3.16–18.09)	4.5 (0.88–9.12)	0.021

*p-value < 0.05

Abbreviations: *MMSE*, Mini-Mental State Examination; *MoCA*, Montreal Cognitive Assessment; *AD-R*, Anxiety and Depression Short Scale; *NPI*, Neuropsychiatry Inventory Scale

**Fig. 1:**
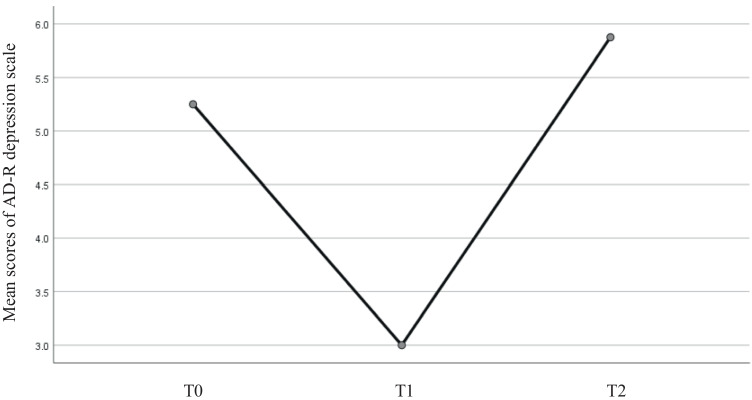
The trend of mean scores of the AD-R depression scale (*N* = 8)

One-way repeated measures ANOVAs were conducted to determine whether there were significant differences in neuropsychological, psychological, and behavioral tests over the course of the rehabilitation program and after 3 months. The assumption of sphericity was met, as assessed by Mauchly’s test of sphericity. No significant differences were found over time in MMSE [*F*(2, 6) = 0.836, *p* > 0.05; *η*_*p*_^2^ = 0.09] and MoCA total scores [*F*(2, 6) = 1.517, *p* > 0.05, *η*_*p*_^2^ = 0.34]. As regard to the psychological scores, no significant changes were found in anxiety score at AD-R scale, [*F*(2, 6) = 1.492, *p* > 0.05, *η*_*p*_^2^ = 0.33], whereas a significant variation in AD-R depression score emerged over time [*F*(2, 6) = 14.93, *p* < 0.01; *η*_*p*_^2^ = 0.83]. Post hoc analysis with Wilcoxon rank-test indicated that depression score at AD-R significantly decreased from T0 to T1, with a reduction from 4.33 ± 3.00 to 3.00 ± 3.16 (*z* =  − 2.304, *p* = 0.021). Furthermore, a significant increase was found from T1 to T2 (follow-up) from 3.00 ± 3.16 to 5.88 ± 4.32 (*z* =  − 2.371, *p* = 0.018), but not from T0 to T2, with a not significant increase (*z* =  − 0.940, *p* = 0.347) — see Fig. 1 for the trend of AD-R depression scores at the different testing time.

Regarding behavioral alterations, analysis of variance did not show any significant difference in NPI scores among T0, T1, and T2, [*F*(2, 6) = 3.17, *p* > 0.05; *η*_*p*_^2^ = 0.51].

## Discussion

To date, few studies have evaluated and tracked over time cognitive, psychological, and behavioral domains of COVID-19 patients. In the present study, we observed the trend of cognitive, psychological, and behavioral variables in patients recovering from COVID-19 in a rehabilitation setting (entrance, T0; discharge, T1) and after a 3-month interval (T2).

The importance of detecting neuropsychological and psychological consequences of this novel coronavirus has been already claimed [3, 11]. This is relevant in order to acquire more knowledge on the possible side effects of COVID-19 and to plan appropriate cognitive rehabilitation and psychological support for inpatients. Moreover, the evaluation of cognitive, behavioral, and psychological conditions is crucial for decisions on adequate post-hospitalization setting patients.

Our data, in accordance with the current literature, show that more than 50% of examined patients provided a poor cognitive performance at screening tests at T0. The finding of behavioral alterations in five out of twelve patients seems to support the necessity to monitor behavioral domains about possible presence of changes in eating, sleep, or usual behavior (i.e., onset of emotional lability, irritability). Moreover, delirium might be present in post-COVID patients [17]. In the present study, only two patients exhibited at T0 delusions and hallucinations. Otherwise, the presence of psychological distress emerged from AD-R scale in our sample (33.3% showed anxiety and 16.6% depression). These data should be taken into account to underline the importance of offering psychological support during the recovery of post-COVID patients.

In the post-acute phase of the disease, patients who have suffered from COVID-19 are very vulnerable concerning both their physical and mental health. This seems to be ascribed to multiple factors, such as prolonged immobilization, isolation [37, 38] from their loved ones and from health workers because of the risk of infection, having experienced the risk of dying, and feeling the uncertainties related to the few knowledge about novel coronavirus.

Assessment of post-COVID patients must take into account that they are living with physical and emotional trauma. Our choice of brief and easily administrable tests at T0 has been mainly due to patients’ physical and psychological weakness and to setting adjustment linked to infection risk. We chose two neuropsychological screening tests in order to have the possibility to detect possible global cognitive impairment with the MMSE or prevalent dysexecutive syndrome with MoCA [15]. Screening tests are not exhaustive to a complete neuropsychological assessment, but they express global cognitive functioning that can be tracked over time and they proved not too stressful for our patients. A similar choice has been made in a recent study [12] where the authors tested COVID-19 patients through the MMSE for the global cognitive functioning, and the FAB to evaluate executive functions. Furthermore, Almeria et al. [14] administered to their patients Global Cognitive Index, and a selected set of tests for memory, attention and executive functions, mental flexibility, and phonemic fluency. Our choice of tests for the analysis of cognitive profile at T2 seems to be appropriate and in line with the rationale of colleagues.

No significant differences were found over time to the screening test (MMSE or MoCA), but between T0 and T1, the mean scores at MoCA showed a slight difference, which on a qualitative level could be representative of a greater deficit in the executive domains or greater sensitivity of the instrument, as suggested by Ortelli et al. [15]. Our finding of poor performance at SDMT in about 62.5% of patients at T2 is in accordance with the data of Zhou et al. [13] who found poor performance of their patients at the Continuous Performance Test (CPT) and seem to show a prolonged impairment of selective and sustained attention in post-COVID patients. According to the literature, abnormal behavior (i.e., hallucinations) could also be present in post-COVID patients [17]. The administration of a scale to detect behavioral alterations as the NPI, therefore, seems to be appropriate. In addition, AD-R Scale has been selected being a psychometric instrument developed to identify clinically significant conditions of distress in patients with cardiac and/or pulmonary disease [35]. We consider important to include these questionnaires during an appropriate psychological interview that is important to know the patient and to capture the presence of psychological distress.

We chose not to evaluate the presence of post-traumatic stress disorder (PTSD) at T0, T1, and T2, considering the possibility of the onset of this disorder later in the recovery phase [39]. A further evolution of our study will be to perform a long-term cognitive and behavioral assessment, approximately 6 months after T2. The possible presence of PTSD will be then evaluated towards an appropriate questionnaire, like IES-R (Impact of Event Scale-Revised) [40].

Our results show an improvement of NPI and AD-R depression scores during hospitalization. The reduction of behavioral alterations between T0 and T1 may be due to the natural evolution of clinical features and to specific physical, psychological, and cognitive improvement due to the rehabilitative training. Moreover, the rehabilitative setting is usually perceived as protective and supportive, promoting emotional and behavioral as well as a physical enhancement.

The increase of depressive symptoms between T1 and T2 may be related to the emotional reactions of these patients when they get back to face the difficulties of current daily living possibly experiencing feelings of uncertainty and fear.

Our experience seems to support the need for an adequate assessment of cognitive, behavioral, and psychological variables in post-COVID patients.

## Limitations

The present study has some limitations, the major one being the small sample size, which does not allow a generalization of our results and limits the possibility of controlling data with regard to other clinical or socio-demographic variables. Dropout is a possible limitation of any study, especially if it consists of a longitudinal research.

Moreover, another limitation of this study is the heterogeneity of our sample: due to the different neurological backgrounds of these patients, this does not allow us to clearly explain the specific impact of COVID-19. Further research will allow us to overcome these limitations and further investigate the neuropsychological sequelae in these patients.

## Conclusions

A cognitive, psychological, and behavioral assessment of post-COVID patients living with physical and psychological vulnerability after having suffered a relevant trauma seems to be necessary. In particular, at the entrance in the Rehabilitation Unit, a global assessment is essential in order to plan the recovery program; meanwhile, assessments at discharge and at the follow-up visit are relevant to gain knowledge about possible long-term neuropsychological and psychological consequences of the COVID-19, and they are mandatory to decide post-hospitalization setting and to organize further support and assistance needs. The choice of assessment tools should take into account the vulnerability of these patients in the immediate post-acute phase of the disease, but it does not rule out the possibility of detailed assessment at follow-up visits, when they usually have recovered part of their physical capacity. In conclusion, we believe that this exploratory study could be a proposal for an assessment model of patients recovering from COVID-19 even if the discussion about times and tools for the evaluation of cognitive, behavioral, and psychological alterations in COVID-19 patients is still open to debate.
